# Single-Cell Transcriptome Analysis of H5N1-HA-Stimulated Alpaca PBMCs

**DOI:** 10.3390/biom13010060

**Published:** 2022-12-28

**Authors:** Menghua Lyu, Xuyang Shi, Yang Liu, Hongyan Zhao, Yue Yuan, Run Xie, Ying Gu, Yuliang Dong, Meiniang Wang

**Affiliations:** 1 BGI-Shenzhen, Shenzhen 518083, China; 2 College of Life Science and Agronomy, Zhoukou Normal University, Zhoukou 466001, China

**Keywords:** alpaca, single cell sequencing, transcriptome, PBMCs, H5N1

## Abstract

Avian influenza A virus H5N1 is a highly pathogenic and persistently a major threat to global health. Vaccines and antibodies targeting hemagglutinin (HA) protein are the primary management strategies for the epidemic virus. Although camelids possess unique immunological features, the immune response induced by specific antigens has not yet been thoroughly investigated. Herein, we immunized an alpaca with the HA antigen of the H5N1 virus and performed single-cell transcriptome profiling for analysis of longitudinal peripheral blood mononuclear cell (PBMCs) behavior using single-cell sequencing technology (scRNA-seq). We revealed multiple cellular immunities during the immunization. The monocytes continued to expand after immunization, while the plasma cells reached their peak three days after the second antigen stimulation. Both monocytes and B cells were stimulated by the HA antigen and produced cell-type-specific cytokines to participated in the immune response. To our knowledge, this is the first study to examine the HA-specific immunological dynamics of alpaca PBMCs at the single-cell level, which is beneficial for understanding the anti-viral immune system and facilitating the development of more potent vaccines and antibodies in camelid animals.

## 1. Introduction

The highly pathogenic avian influenza virus (HPAIv) has caused worldwide epidemics in poultry and humans [1]. The H5N1 influenza virus first affected humans in 1997; in 2021, it spread at an unprecedented rate throughout the east coasts of Canada and the United States. Since late 2003, H5N1 has become the most contagious and deadly pathogen in domestic fowl and wild birds, as well as in human populations in Asia, the Middle East, Eastern Europe, and Africa. Over 800 human cases have been recorded, with a fatality rate of more than 50% [1,2]. Hemagglutinin (HA) is a major envelope glycoprotein on the surface of the influenza virus. By mediating the fusion of the endosomal membrane through interaction with the sialic acid receptors on target cells, the HA trimer initiates the viral infection [3,4,5,6]. Therefore, HA proteins have been utilized as primary targets for the influenza vaccine and antibody development to neutralize the influenza virus. Therapeutic approaches that interfere with the HA protein on the influenza virus have demonstrated excellent anti-viral activity in clinical trials [3,7,8].

Camelids as domesticated animals are significant for the economy in many regions of the world and can adapt to a wide range of extreme ecosystems [9]. Recent research on camelid B cells has shown that all individuals of this species possess a special immunological trait that is uncommon in other species [10]. A ‘nanobody’ is a novel single-domain antibody derived from the variable domain of the heavy chain, from heavy-chain-only antibodies (VHH) in camelids. Due to the unique biochemical characteristics, including small size, high affinity, low cytotoxicity, as well as deep tissue penetration [11], nanobodies have been considered the “next generation” antibodies [12]. Recently, there has been an increased interest in camelid immunology. Considerable research has been devoted to studying immunoglobulin and nanobody development [13,14,15]. However, the immune system consists of a complex network of immune cells and related cytokines that contribute to efficient immune responses against infections. Studies on the immune response to antigen-specific immunization are important to understand the immune system of camelid animals.

To date, only a few studies have investigated the cellular components of the camelid immune system. Several studies have described the diversity of peripheral blood mononuclear cells (PBMCs) and revealed the relative proportions of the primary leukocyte subpopulations in camelid animals using flow cytometry [9,16]. Due to the small number of identified cell subgroups and the inconsistent phenotypic and functional definition of leukocyte composition, it is challenging to accurately compare the immunophenotype of leukocytes obtained in different studies [17,18,19]. Single-cell RNA sequencing (scRNA-seq) technology offers unprecedented precision for describing novel cell types, cell states varying from healthy to pathological, or immune cell responses to antigen stimulation [20]. Sophisticated scRNA-seq technology has been used to analyze the kinetics of the immune response during pathogen infections and to reveal the immune regulation mechanism at the single-cell level [21].

In this study, we collected PBMCs from an alpaca and described the transcriptome landscape and longitudinal alterations in response to H1N1-HA protein immunization at the single-cell level. A total of 35,853 cells were obtained during the pre- and post-immunization stages. Based on transcriptome data, we defined the immunophenotype of leukocytes and discovered that HA antigen boosting triggered both innate and adaptive immune responses in the alpaca. Characterization of important lymphocyte subpopulations and dynamics of the immune response to antigen-specific immunization offer valuable information for the development of potent nanobodies and influenza vaccines in camelid animals.

## 2. Materials and Methods

### 2.1. Alpaca Immunization

**Figure 1:**
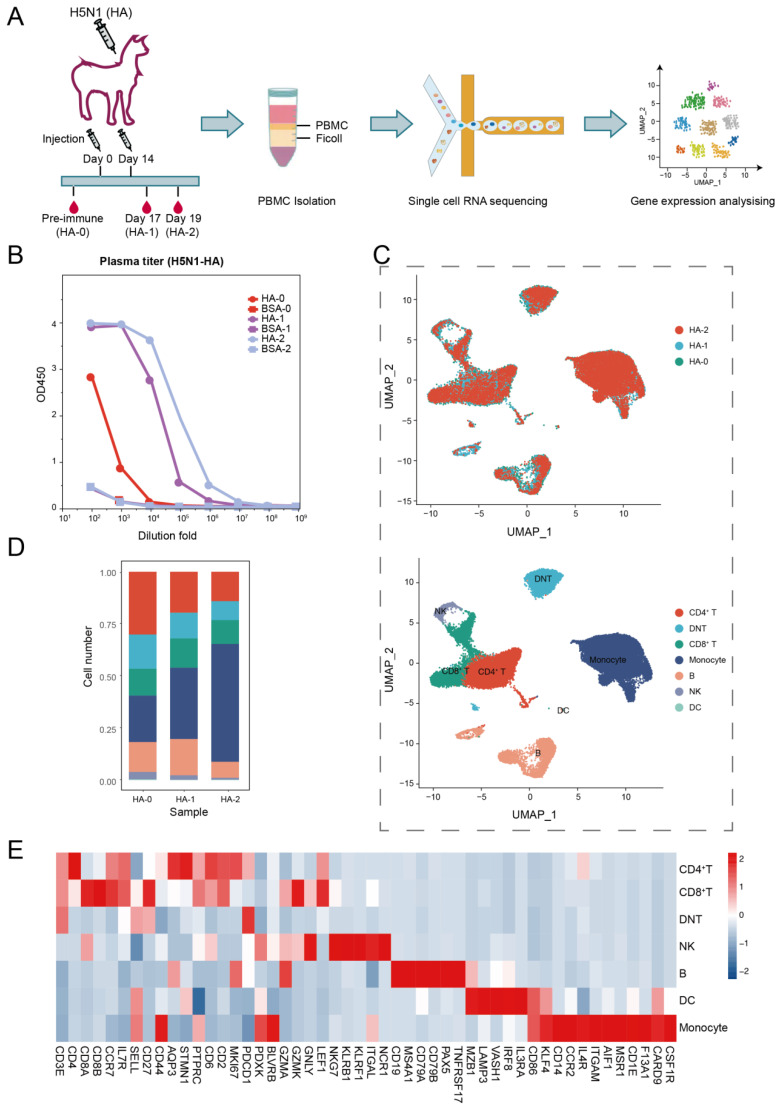
Study design and single-cell dynamic landscape of HA-stimulated PBMCs in the alpaca. (**A**) The alpaca received HA stimulations two times, and blood samples were taken from the alpaca at three time points before and after stimulations. Then, scRNA-seq dynamic analyses were conducted. (**B**) Line plots display the result of the ELISA binding assay testing the titer of the HA-specific antibody in alpaca serum. (**C**) Top: UMAP plot shows HA-0, HA-1, and HA-2 in three colors indicating no batch effects; Bottom: UMAP plot shows seven major cell types of 35,853 immune cells by unsupervised clustering. Cells are colored by clusters. (**D**) Histogram shows the cell proportion of PBMC at different time points. (**E**) Heatmap shows representative marker expression patterns, which annotate clusters to related cell types.

A young, healthy adult female alpaca raised in the China National Gene Bank was immunized twice over a period of 14 days. For immunization, the animal was injected with 250 µg of influenza A hemagglutinin protein (HA1-V5229-1mg, ACROBiosystems, Newark, DE, USA) mixed 1:1 with complete/incomplete Freund’s adjuvant. Whole blood samples were collected before immunization (day 0, HA-0), and at day 17 (HA-1) and day 19 (HA-2) after the second immunization (Figure 1A).

### 2.2. Serum Titer Assay

An HA-specific enzyme-linked immunosorbent assay (ELISA) binding assay was performed to determine the antibody titer in the alpaca serum. Maxisorp ELISA plates (Invitrogen) were coated with 100 ng of HA protein in a coating buffer (100 nM NaHCO_3_ in phosphate-buffered saline (PBS), pH 8.3) and incubated overnight at 4 °C, with corresponding blank (non-antigen containing) control and negative (irrelevant antigen containing) control sets. Next, 200 μL of blocking buffer (2% skimmed milk in PBS) was added for 1 h at 25 °C. The alpaca serum collected at HA-0, HA-1, and HA-2 was serially diluted from 10^1^- to 10^8^-fold with phosphate-buffered saline (PBS) and incubated in the plate at 25 °C for 1 h. Next, the plates were washed five times with PBST (0.05% Tween-20 in PBS) before adding the secondary antibody. HRP-conjugated mouse anti-alpaca IgG-antibody (Abcam) was added at a 1:2000 dilution and incubated for 1 h at 25 °C. After adding 100 μL TMB substrate (Abcam, Cambridge, UK), the absorbance at 450 nm was read within 15 min using Synergy™ H1 (BioTek, Winowski, VT, USA).

### 2.3. Peripheral Blood Mononuclear Cell (PBMC) Isolation

Whole blood samples were collected, and peripheral blood mononuclear cells (PBMCs) from each sample were isolated using Ficoll-Paque™ PLUS Media (GE Healthcare, Chicago, IL, USA) within 2 h, according to the manufacturer’s instructions. Briefly, 10 mL of whole blood was transferred from the collection vessel to a 50 mL EP tube; an equal volume of PBS solution was added to the EP tube to dilute the blood. Then, the diluted blood was added to 15 mL of Ficoll and centrifuged at 500× *g* for 20 min (accelerate 3, decelerate 0); next, the buffy coat was carefully transferred to a new tube and diluted with PBS to a total volume of 20 mL. The samples were centrifuged again at 600× *g* for 6 min, after which the buffer was discarded, and the cell pellet was resuspended in 3 mL PBS.

### 2.4. Single-Cell Library Construction and Sequencing

The DNBelab C Kit (MGI, #1000021082) was used to construct the library. Isolated PBMCs were resuspended as single cells at a density of 5000 cells/mL. Cells were wrapped in droplets with a negative pressure chip, and mRNA was transcribed to cDNA to generate a sequencing library according to the manufacturer’s instructions. Sequencing libraries were quantified using a Qubit™ ssDNA Assay Kit (Thermo Fisher Scientific, Waltham, MA, USA). The cDNA libraries were then subjected to DIPSEQ T1 sequencing (MGI).

### 2.5. scRNA-Seq Data Processing

High-quality scRNA-seq data with valid barcodes were aligned to the genome of VicPac3.1 through the STAR software, and a unique molecular identifier (UMI) count matrix was generated using PISA (version 1.10.2) (https://github.com/shiquan/PISA (accessed on 8 August 2021)). The raw transcript count matrix was loaded into the R (v4.0) software using the Seurat (v3.1.5) package [22].

### 2.6. Data Integrating and Cell Clustering

We used the R package Seurat 3.1.5 to integrate and analyze datasets from the three samples (HA-0, HA-1, and HA-2). The integrated mRNA expression matrix was first filtered following the Seurat recommendation and a total of 35,853 cells with unique UMI were obtained [23]. Unsupervised clustering was conducted using Seurat with the parameter res = 0.5, which revealed a total of 18 clusters. We used mRNA biomarkers obtained from recently published articles to classify these clusters into seven major groups (Supplemental Table S1) [24].

### 2.7. Differentially Expressed Gene (DEG) Analysis

DEGs were calculated using the function *FindMarkers* built in *Seurat* with the default parameters. The resulting DEGs were filtered with p_val_adj < 0.05 and then sorted according to the average log2 fold change (avg_log2FC).

### 2.8. Gene Ontology Analysis

We used the *clusterProfiler* to annotate the functions of the cell subsets. We filtered the enriched pathways with an FDR q-val of ≤0.05 [25]. The pathways that normalized the enrichment score in the top 20 are shown in the results.

## 3. Results

### 3.1. Overview of the Transcriptome Landscape of Alpaca PBMCs

To determine how immunization with the HA protein affected the transcriptome landscape of the alpaca immune system, we administered an adult alpaca twice with the purified HA protein of the H5N1 virus on day 0 and day 14. Using a DIPSEQ T1 sequencer, we sequenced and analyzed the PBMCs isolated on day 0 (HA-0) prior inoculation and days 17 (HA-1) and 19 (HA-2) after the second inoculation (Figure 1A). The serum titer assay revealed that after the second immunization, humoral immunity was boosted by HA-specific antibodies in the alpaca (Figure 1B).

Following strict quality control (with > 350 genes per cell), PBMCs at HA-0, HA-1, and HA-2 generated 13,284, 10,792, and 11,769 single cells, respectively. The average numbers of genes per cell for the three samples were 825, 1163, and 1355, respectively (Supplemental Figure S1). The integrated 35,853 cells were analyzed and plotted using uniform manifold approximation and projection (UMAP) (Figure 1C). Unsupervised clustering of the integrated PBMCs yielded seven major clusters (Figure 1C,E) according to the canonical markers listed in Supplemental Table S1, including CD4^+^ T cells (*CD3*^+^, *CD4*^+^, *CD8*^−^), CD8^+^ T cells (*CD3*^+^, *CD4*^−^, *CD8*^+^), double naive T cells (DNT, *CD3*^+^, *CD4*^−^, *CD8*^−^), monocytes (CD3^−^, *CD14*^+^, *ITGAM*^+^, *CD86*^+^, *KLRF4*^+^), B cells (*CD19*^+^, *MS4A1*^+^, *CD79A*^+^, CD79B^+^), natural killer cells (NK, *CD3*^−^, *NKG7*^+^, *KLRB1*^+^), and dendritic cells (DC, *MZB1*^+^, *LAMP3*^+^, *VASH1*^+^, *IRF8*^+^, *IL3RA*^+^, *CD86*^+^, *KLF4*^+^). Additionally, we compared the leukocyte composition between HA-0, HA-1, and HA-2 (Figure 1D, Supplemental Table S2). The immune cell compositions of alpacas underwent a significant alteration upon HA antigen inoculation. Particularly, the percentage of monocytes dramatically increased from 22.31% at baseline (HA-0) to 56.60% on day 19 (HA-2), which implies that innate immunity is involved in antigen-specific immunity.

### 3.2. Monocyte Response to HA Immunization

**Figure 2:**
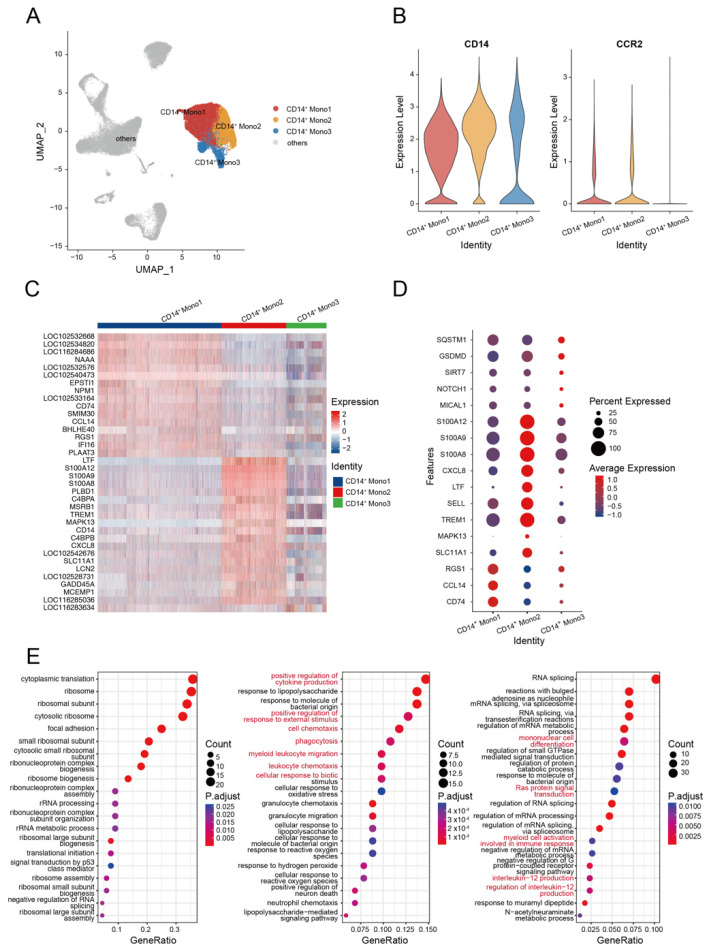
Examination of subclusters of monocyte cells in HA-stimulated PBMCs. (**A**) UMAP plot shows the results of sub-clustering involving monocyte cells. (**B**) The classic marker gene was identified in three clusters. (**C**) Heatmap shows differential gene expression patterns in various monocyte cell types. (**D**) Dotplot shows the marker gene expressions pattern in various clusters. (**E**) Representative GO terms and pathways enriched in DEGs of various monocyte cell subsets of PBMCs. Left, CD14^+^ Mono1; middle, CD14^+^ Mono2; right, CD14^+^ Mono3.

Monocytes are crucial immunological components that play important roles in infection resistance. They participate in innate immune reactions and serve as a bridge to adaptive immune reactions [26]. Based on the relative expression levels of *CD14* and *CCR2* (*CD16* was not found in the current study), we divided the alpaca monocytes into three subsets (Mono1, Mono2, and Mono3) to describe their functional characteristics. We identified the *CCR2* gene in the Mono1 and Mono2 subsets (*CCR2*^+^), but not in the Mono3 subset (*CCR2*^−^). The Mono2 subset was differentiated from Mono1 by a higher expression level of *CD14* (Figure 2A,B, Supplemental Figure S2). According to the heatmap, alpaca monocytes constituted heterogeneous populations with distinct transcriptional profiles (Figure 2C).

Mono1 is the majority subset of monocytes, accounting for over 50% of the total monocytes (Supplemental Table S3). Mono1 shows *CD14*^+^, *SELL*^+^, *IL4R*^+^, *ITGAL*^+^, *ITGAM*^−^, *AIF1*^+^, *PTPRC*^+^, *CD44*^+^, *MSR1*^+^, *F13A1*^+^, *CARD9*^+^, *PDXK*^+^, *CSF1R*^+^, and *BLVRB*^+^. The Mono2 subset shows *CD14*^+^, *SELL*^+^, *IL4R*^+^, *ITGAL*^+^, *ITGAM*^+^, *AIF1*^+^, *PTPRC*^+^, *CD44*^+^, *MSR1*^+^, *F13A1*^+^, *CARD9*^+^, *PDXK*^+^, *CSF1R*^+^, and *BLVRB*^+^. Compared with Mono2 and Mono3 subsets, Mono1 cells highly expressed the MHC class II antigen-related gene *CD74* and chemotaxis-related gene *CCL14* and *RGS1*. Mono2 cells highly expressed genes related to cell activation and differentiation (such as *SLC11A1* and *MAPK13*) [27,28], cell trafficking (*TREM1* and *SELL*) [29], cytokine production *LTF* [30], and chemokine ligand *CXCL8*, which indicate the pro-inflammatory state of monocytes. Intriguingly, we discovered that Mono2 cells had high levels of expression of S100 protein-coding genes, including *S100A8*, *S100A9*, and *S100A12* (Figure 2D), which are associated with inflammatory processes [31,32,33]. Mono3 cells show *CD14*^+^, *SELL*^+^, *IL4R*^−^, *ITGAL*^+^, *ITGAM*^−^, *AIF1*^+^, *PTPRC*^−^, CD44^−^, MSR1^−^, *F13A1*^+/−^, *CARD9*^−^, *PDXK*^−^, *CSF1R*^−^, and *BLVRB*^−^. The Mono3 subset highly expressed the reactive oxygen species (ROS) production modulation gene *MICAL1* [34], cell tracking and/or differentiation-related gene *NOTCH1* [35,36,37] and *SIRT7* [38], and the inflammation markers *GSDMD* and *SQSTM1* [39,40].

Next, we conducted a gene ontology (GO) analysis of the upregulated genes for each of the three monocyte subsets. Significant enrichments in the pathways of the positive regulation of cytokine production, positive regulation of response to external stimulus, cell chemotaxis, phagocytosis, myeloid leukocyte migration, cellular response to biotic stimulus, and leukocyte chemotaxis were identified in Mono2 cells (Figure 2E). These findings indicate that the Mono2 subset of alpaca is similar to classical human monocytes and participates in the pro-inflammatory process by activating and priming phagocytosis, innate sensing/immune responses, and migration [41]. Genes highly expressed in Mono3 cells were significantly enriched in mononuclear cell differentiation, Ras protein signal transduction, myeloid cell activation involved in immune response, interleukin-12 production, and regulation of interleukin-12 production (Figure 2E), suggesting that these processes were triggered by HA immunization, resulting in the production of IL-12.

### 3.3. B Cell Response to H5N1-HA Stimulation

**Figure 3:**
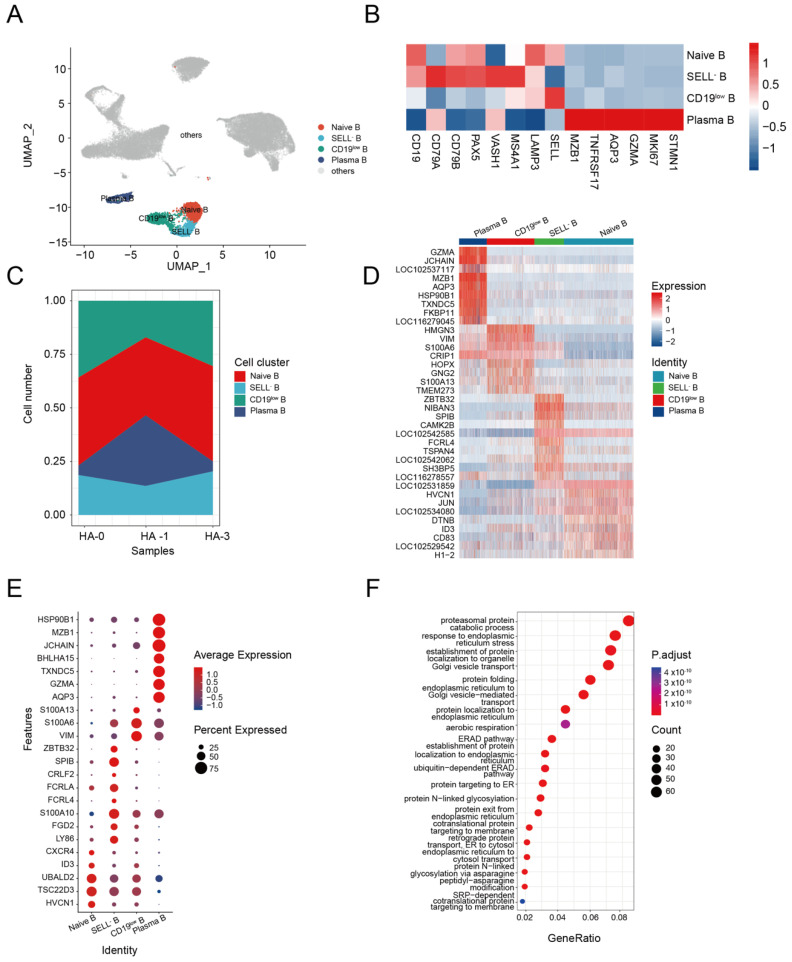
Dynamic changes in functional status among B cell subsets. (**A**) UMAP plot shows the results of sub-clustering of B cells. (**B**) Heatmap shows representative marker expression patterns, annotating clusters to related B cell types. (**C**) Dynamic changes of B cell proportions were identified two times after immunization. (**D**) Heatmap shows differential gene expression patterns in various B cell types. (**E**) Dotplot shows the marker gene expressions pattern in various clusters. (**F**) Representative GO terms and pathways enriched in DEGs of plasma B cell subsets of PBMC.

Humoral immunity mediated by the B cell response is known to protect the host during different pathogen infections [42]. We identified four subsets of B cells from alpaca PBMCs, including naive B cells (*CD19^+^*, *SELL^+^*), SELL^−^ B cells (*SELL^−^*, *MS4A1^+^*, *LAMP3^+^*), CD19^low^ B cells (CD19^low^, *MS4A1^+^*), and plasma B cells (*MZB1^+^*, *GZMA^+^*, *AQP3^+^*) (Figure 3A,B). The composition and changes of the four subsets at HA-0, HA-1, and HA-2 are shown in Figure 3C. The plasma B cell composition of total B cells changed from 4.65% at HA-0 to 32.96% at HA-1 and then back to 4.65% at HA-2 (Supplemental Table S4). Given the production of HA-specific antibodies in the serum (Figure 1B), we assumed that plasma B cells were involved in the enhancement of immunity during immunization with the HA antigen.

Using the *FindAllMarkers* function, we profiled the transcriptome characteristics of naive B, SELL^−^ B, CD19^low^ B, and plasma B cell subsets and identified differentially expressed genes (DEGs) (Supplemental Table S5). The heatmap of the top 10 DEGs revealed that the four B-cell clusters were distinctly differentiated from each other (Figure 3E).

DEG analysis revealed that the naive B cells highly expressed genes involved in homeostasis, such as *HVCN1*, *TSC22D3* (*GILZ*), *UBALD2* gene, transcription factor *ID3* molecule, and the chemokine receptor *CXCR4*, indicating a relative resting state [43,44,45] (Figure 3F). SELL^−^ B cells highly expressed a series of genes related to memory B (*LY86*, *FGD2*, and *S100A10*) [46,47]. However, some genes related to the atypical memory B cell phenotype, such as the Fc family receptor genes *FCRL4* and *FCRLA*, *CRLF2* [48], *SPIB* [49], and *ZBTB32* [50], were found in the SELL^−^ B cells (Figure 3F). These results indicated that the SELL^−^ B cells are dysfunctional atypical memory B cells. CD19^low^ B cells highly expressed genes related to cell motility, such as the encoding type III intermediate filament protein (*VIM*) [51] and S100 calcium-binding proteins (*S100A6*, *S100A13*) [52,53], indicating their activation state (Figure 3F). Plasma B cells highly expressed genes related to cell activation (*AQP3*, *GZMA*) [54], differentiation (*TXNDC5*, *BHLHA15*) [55,56], and antibody production (*JCHAIN*, *MZB1*, and *HSP90B1*) (Figure 3F) [57,58,59,60,61]. GO analysis demonstrated the significant enrichment of genes associated with Golgi vesicle transport, protein folding, endoplasmic reticulum to Golgi vesicle-mediated transport, protein localization to endoplasmic reticulum, and establishment of protein localization to endoplasmic pathways in plasma B cells (Figure 3G). These results suggest that repeated HA immunization could activate alpaca B cells, which produce antibodies to induce a humoral immune response against the infection.

### 3.4. The Transcriptome Landscape of T Cells in the Alpaca

**Figure 4:**
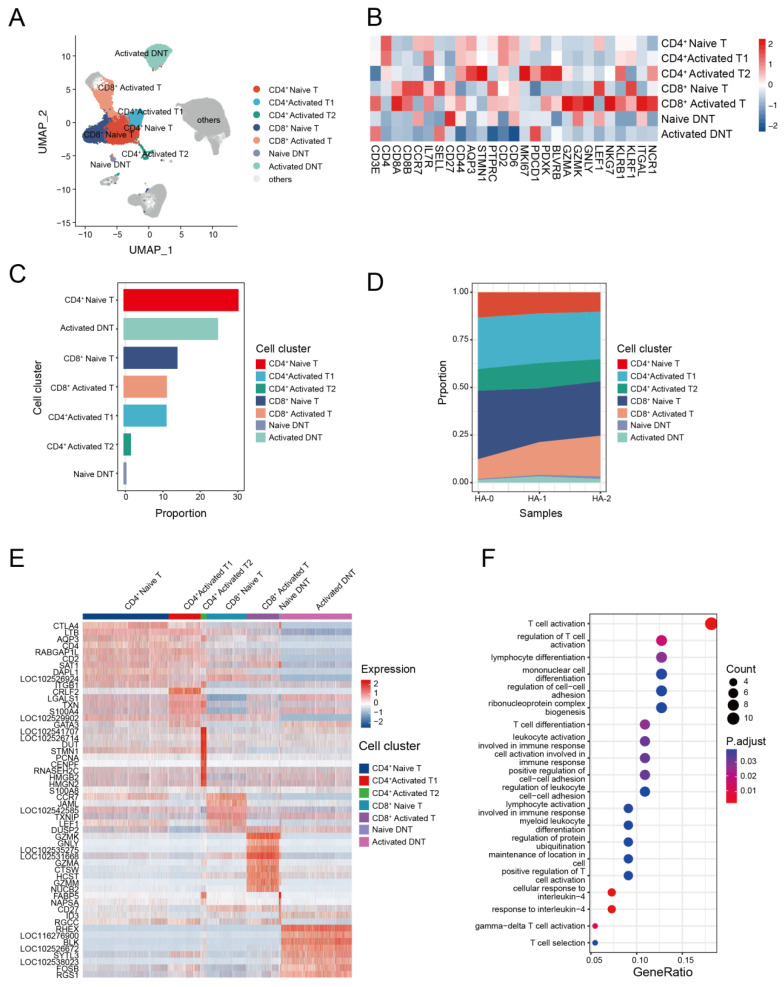
T cell immunity in response to H5N1-HA stimulation. (**A**) UMAP plot shows the results of sub-clustering of T cells. (**B**) Heatmap shows representative marker expression patterns, annotating clusters to related T cell types. (**C**) Histogram shows PBMC cell proportions in various T cell types. (**D**) Dynamic changes in T cell proportions were identified two times after immunization. (**E**) Heatmap shows differential gene expression patterns in various T cell types. (**F**) Representative GO terms and pathways enriched in DEGs of CD8^+^ activated T subsets of PBMCs.

T cells play a key role in the immune response against avian IAV infections [62,63]. To investigate the transcriptome characteristics of T cells, we further classified the T cell subsets with canonical markers into seven clusters. These included three CD4^+^ T cell subsets: CD4^+^ naive T cells (*CD4*^+^, *CCR7*^+^), CD4^+^ activated T1 cells (*CD4*^+^, *KLRB1*^+^), and CD4^+^ activated T2 cells (*STMN1*^+^, *MKI67*^+^, *PDCD1*^+^); two CD8^+^ T cell subsets: CD8^+^ naive T cells (*CD8A*^+^, *LEF1*^+^, *CCR7*^+^) and CD8^+^ activated T cells (*CD8A*^+^, *GZMK*^+^); and two double negative T (CD4^−^CD8^−^ T, DNT) cell subsets: naive DNT (*CD27*^+^) and activated DNT (*PDCD1^+^*) (Figure 4A,B, and Supplemental Figure S3).

The compositions of the seven T cell subsets revealed that CD4^+^ T cells were the dominant T cell subset (Figure 4C and Supplemental Table S6). Analysis of T cell composition at the sample level revealed a significant decline in naive CD4^+^ T cells and an increase in activated CD8^+^ T cells following the second immunization. This finding suggests that antigen immunization may stimulate the proliferation of activated CD8^+^ T cells (Figure 4D). To identify cell-subtype-specific gene signatures associated with antigen stimulation, we performed an integrated comparative analysis of DEGs from T cell subgroups and found that alpaca T cells exhibited heterogeneous transcriptional changes (Figure 4E). Naive CD4^+^ T cells highly expressed *CCR7* and *LEF1*, and activated CD4^+^ T cells expressed *S100A4* and *S100A8*, while *GZMK*, *GNLY*, and *GZMA* genes were enriched in activated CD8^+^ T cells. GO analysis revealed that the up-regulated expression genes in activated CD8^+^ T cells were mostly involved in T cell activation, cell killing, and the regulation of the immune effector process. Upregulated genes in other T cell subsets were enriched in different pathways and were associated with various function (Figure 4F).

## 4. Discussion

Studies on camelid immunity have mainly focused on the generation of nanobodies, which have been widely used in therapeutics and diagnostics [64,65]. Few studies have examined the characteristics of alpaca immune cell composition in response to antigen immunization. In this study, we constructed a comprehensive single-cell landscape of peripheral immune cells from an alpaca with HA antigen stimulation. Using single-cell RNA sequencing, we profiled 35,853 immune cells sampled before and after immunizations. Immune cells were classified into seven major clusters, including CD4^+^ T cells, naive T cells, monocytes, natural killer cells, and dendritic cells. The immune responses of PBMC clusters were analyzed in detail separately. It is crucial to systematically identify the characteristics of immune cells in response to antigen-specific immunization, and this dataset will undoubtedly further elucidate the underlying molecular mechanisms of the unique immune system of camelid animals.

Antigen-mediated induction of antigen-specific B and T-cell responses requires the activation of the innate immune system, particularly with respect to antigen-presenting cells [66]. Following HA immunization, we found that the proportion of CD14^+^ monocytes steadily increased to >50%. Two subsets of CD14^+^ monocytes were activated and displayed pro-inflammatory characteristics by upregulating the expression of genes related to cell activation and differentiation, cell trafficking, and cytokine production. DEG analysis revealed that monocytes were mostly enriched in activities of antigen presentation and granulocyte chemotaxis pathways, which indicates that the innate and adaptive immune cells were activated in response to HA stimulation in the alpaca. However, the activation of the innate immune system can be both protective and detrimental during infections [67]. HPAIv infections can induce a cytokine storm and exaggerate innate immune response, which results in severe pneumonia or death [26,62,68,69]. The substantial increase in monocyte percentage and cytokine production seen in response to HA immunization may indicate an excessive infiltration of pro-inflammatory monocytes, which may result in immunopathology [26,70]. This discovery offers a different perspective to illustrate the generation of cytokine storms.

In addition to the monocytes, the proportion of CD8^+^ activated T cells also increased. GO analysis revealed that the T cell activation pathway and expression levels of cytotoxicity-related genes were upregulated in CD8^+^ activated T cells. These results demonstrated that HA-immunization activated the innate immune system, which could assist T cell activation against the infection. We have also noticed that part of the *CCR7*^+^*SELL*^+^ CD4^+^ naive T cell cluster identified in this study simultaneously expressed T cell activating genes such as *CTLA4* and *LTB*. This phenomenon emphasizes that naive T cells are much more heterogeneous than previously understood [71,72,73,74,75]. These *CCR7*^+^*CTLA4^+^* T cells were evenly distributed among the HA-0, HA-1, and HA-2 samples (data not shown), indicating that the expression of *CTLA4* CD4^+^ in naive T cells was not induced by HA immunization. We speculate that the co-expression of *CCR7* and *CTLA4* in these CD4^+^ naive T cells might be a result of continuous stimulation by other antigens several months prior to HA immunization. Further studies on the immune landscape of camelid animals are needed to reveal the occurrence of these naive T cells.

Four B cell subclusters, including naïve B cells, CD19^low^ B cells, SELL^−^ B cells, and plasma B cells were identified in the alpaca PBMCs. This study revealed that SELL^−^ B cells highly express the *FCRL4* gene, which may inhibit memory B cells from differentiating into plasma cells by reducing the proliferation and differentiation potential [76]. SELL^−^ B cells were assumed to have an atypical memory B cell phenotype following specific antigen stimulation [77]. A high proportion of plasma B cells highly expressed antibody secretion-related genes, such as *TNFRSF17* (*BCMA*), *FKBP11*, and *MZB1*, which can be utilized to develop antibodies targeting alpaca plasma B cells. This is helpful for alpaca plasma cell enrichment during nanobody development. The proportion of plasma B cells during antigen immunization determines the effectiveness of antigen-specific antibody generation [78,79,80]. In this study, we found that the proportions of plasma B cells dramatically increased by day 17 but were significantly reduced by day 19 (day 5 after the second HA immunization), suggesting that the proportion of plasma B cells would reach a peak at three days after the second immunization. Plasma cell dynamics during the specific antigen immunization should offer valuable references for nanobody discovery in immunized camelids [79,80]. However, the sampling periods between the first and second immunizations were not studied here. A larger and more thorough cohort of camelid animals should be constructed in the future to accurately identify the molecular mechanisms underlying the immune response.

## 5. Conclusions

In summary, we demonstrated the dynamic characteristics of the immune response in an alpaca following immunization with the H5N1 virus HA protein. To our knowledge, this is the first study to examine the immune cell population in camelids at the single-cell level. Our study revealed that both innate and adaptive immune cells were activated after HA antigen stimulation in the alpaca. This work may offer new insights for understanding the unique camelid immune system and provide benefits for research focused on potent vaccine development and antigen-specific nanobodies.

## Data Availability

The data that support the findings of this study have been deposited into the CNGB Sequence Archive of CNGBdb with accession number CNP0003408 [81,82].

## Supplementary Materials

The following supporting information can be downloaded at: https://www.mdpi.com/article/10.3390/biom13010060/s1; Figure S1. scRNA-seq data quality control of alpaca’s PBMCs in each sampling time; Figure S2. Violin plots show the expression levels of mark genes in each subset of monocyte, Figure S3. Feature plots show the expression levels of mark genes in each T cell subset; Table S1. Canonical markers of cell clusters; Table S2. Composition of cell clusters in each sample; Table S3. Proportations of Monocyte subsets in each sample; Table S4. Proportations of B cell subsets in each sample; Table S5. Differentialy expressed genes among B cell subsets; Table S6. Proportations of T cell subsets in each sample.
