# Supplementary material for: Single-Cell Transcriptome Analysis of H5N1-HA-Stimulated Alpaca PBMCs
Source: Biomolecules. 2022 Dec 28;13(1):60. doi: 10.3390/biom13010060 (PMC9855979; doi:10.3390/biom13010060)
Supplement: Supplementary file 1 [file biomolecules-13-00060-s001.zip › Supplementary Figures.pdf]

Single-cell transcriptome profiling of the HA-specific immune response in the Alpaca

Menghua Lyu[1,2#], Xuyang Shi[1#], Yang Liu[1], Hongyan Zhao[1], Yue Yuan[1], Run Xie[1], Ying Gu[1], Yuliang Dong[1], Meiniang Wang[1*]

1. BGI-Shenzhen, Shenzhen 518083, China
2. College of Life Science and Agronomy, Zhoukou Normal University, Zhoukou 466001, China

[#]These authors contribute equally to this work

***Correspondence:**

Meiniang Wang

wangmeiniang@genomics.cn

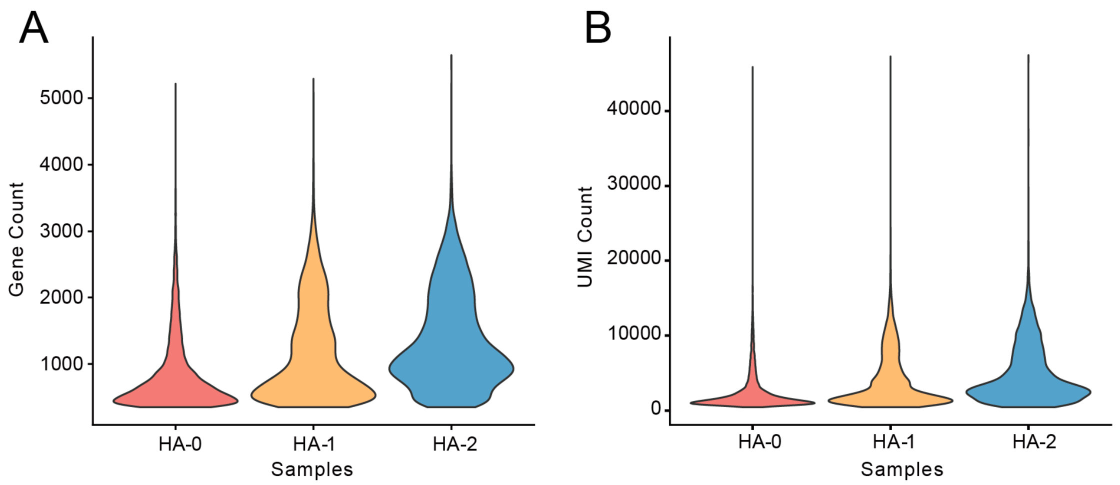

Supplementary Figure S1. scRNA-seq data quality control of alpaca's PBMCs in each sampling time. Violin plot for the distribution of (A) gene number, (B) UMI number.

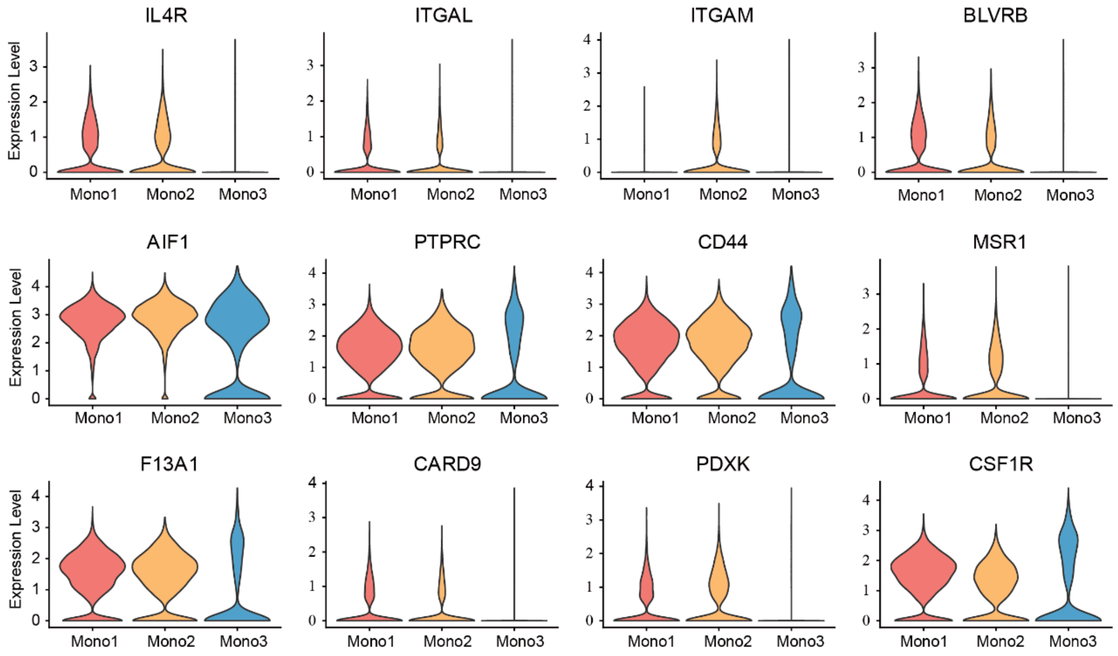

Supplementary Figure S2. Violin plots show the expression levels of mark genes in each subset of monocyte.

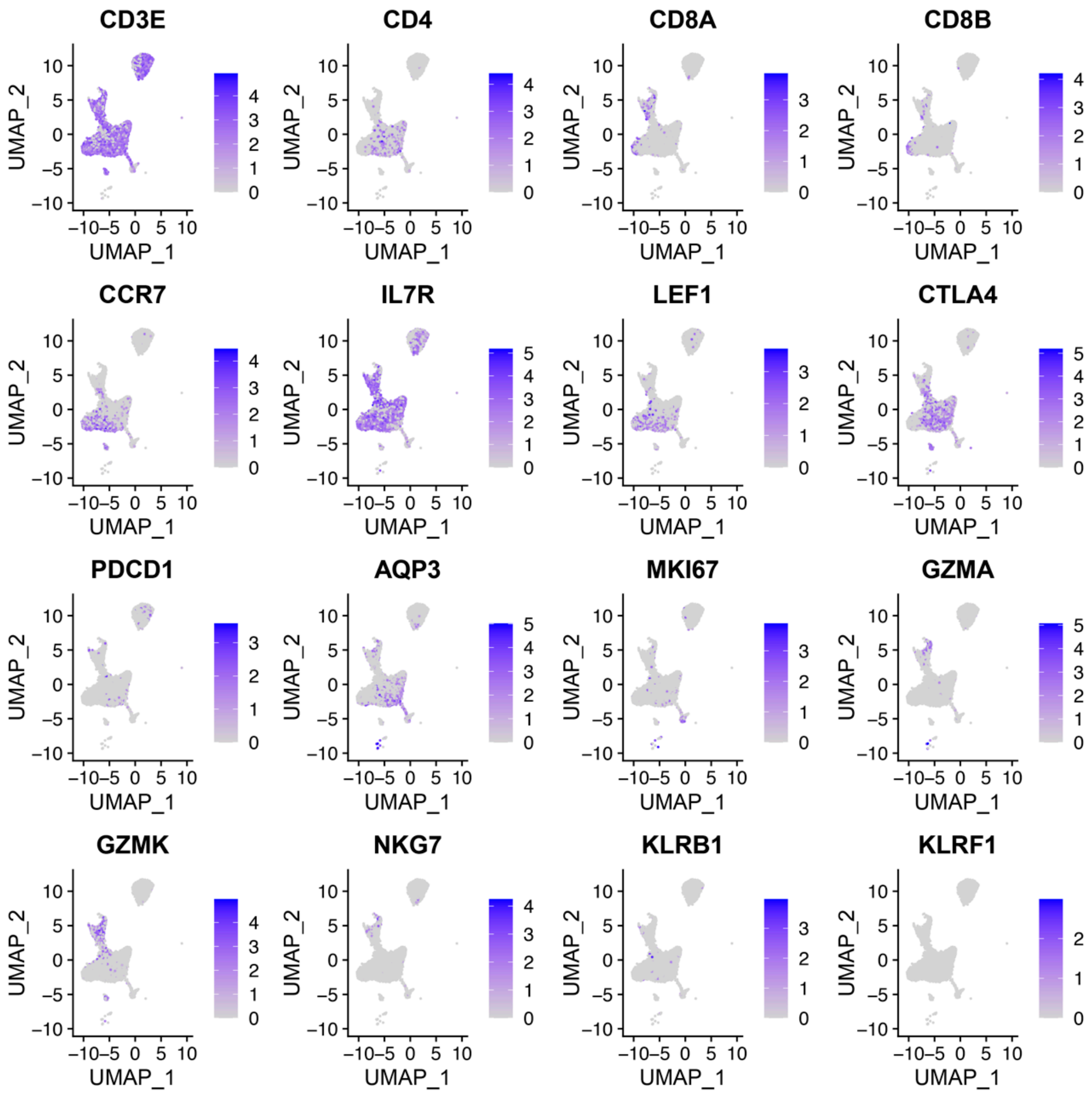

Supplementary Figure S3. Feature plots show the expression levels of mark genes in each T cell subset.
